# LncRNA ZFAS1 promotes invasion of medullary thyroid carcinoma by enhancing EPAS1 expression via miR‐214‐3p/UCHL1 axis

**DOI:** 10.1002/ccs3.12021

**Published:** 2024-04-12

**Authors:** Wenjing Chen, Shaoqing Wang, Dongmei Wei, Lili Zhai, Li Liu, Chunlei Pan, Zhongshu Han, Huiming Liu, Wei Zhong, Xin Jiang

**Affiliations:** ^1^ Department of Pathology Qiqihar First Hospital Qiqihar Heilongjiang Province China; ^2^ Pathology of Qiqihar Medical College Qiqihar Heilongjiang Province China; ^3^ Department of Science and Education Qiqihar First Hospital Qiqihar Heilongjiang Province China; ^4^ Department of CT Radiology Qiqihar First Hospital Qiqihar Heilongjiang Province China; ^5^ Department of General Surgery Qiqihar First Hospital Qiqihar Heilongjiang Province China; ^6^ Department of Critical Care Medicine Qiqihar First Hospital Qiqihar Heilongjiang Province China; ^7^ Qiqihar First Hospital Qiqihar Heilongjiang Province China; ^8^ Department of Orthopaedics Third Affiliated Hospital of Qiqihar Medical College Qiqihar Heilongjiang Province China

**Keywords:** EPAS1, invasion, lncRNA ZFAS1, medullary thyroid carcinoma, miR‐214‐3p

## Abstract

lncRNA ZFAS1 was identified to facilitate thyroid cancer, but its role in medullary thyroid carcinoma (MTC) remains unknown. This study aimed to unravel the potential function of this lncRNA in MTC by investigating the involvement of the lncRNA ZFAS1 in a ceRNA network that regulates MTC invasion. Proliferation, invasion, and migration of cells were evaluated using EdU staining and Transwell assays. Immunoprecipitation (IP) assays, dual‐fluorescence reporter, and RNA IP assays were employed to examine the binding interaction among genes. Nude mice were used to explore the role of lncRNA ZFAS1 in MTC in vivo. ZFAS1 and EPAS1 were upregulated in MTC. Silencing ZFAS1 inhibited MTC cell proliferation and invasion under hypoxic conditions, which reduced EPAS1 protein levels. UCHL1 knockdown increased EPAS1 ubiquitination. ZFAS1 positively regulated UCHL1 expression by binding to miR‐214‐3p. Finally, silencing ZFAS1 significantly repressed tumor formation and metastasis in MTC. LncRNA ZFAS1 promotes invasion of MTC by upregulating EPAS1 expression via the miR‐214‐3p/UCHL1 axis.

## Graphical abstract

lncRNA ZFAS1 promotes EPAS1‐mediated invasion in medullary thyroid carcinoma through the ceRNA network of ZFAS1/miR‐214‐3p/UCHL1.

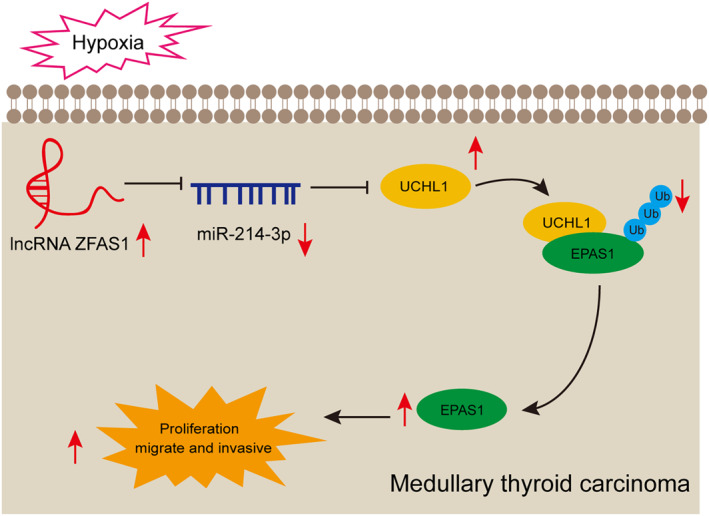

## 1 INTRODUCTION

Medullary thyroid carcinoma (MTC) is a subtype of thyroid cancer (TC) that was first discovered and characterized in 1959.^1^ The prevalence of MTC gradually increased.^2^ Although localized MTC can be effectively controlled through advanced surgery, it frequently exhibits a high rate of relapse, and a considerable proportion of patients experience primary metastasis.^3^ Therefore, it is imperative to conduct further investigations into the mechanisms underlying MTC invasion and metastasis.

Hypoxia is a pervasive feature of the tumor microenvironment in different types of tumors, and it plays a crucial role in the invasion and metastasis of nearly all solid tumors, including MTC.^4^ Endothelial PAS domain‐containing protein 1 (EPAS1), also known as hypoxia‐inducible factor‐2 alpha (HIF‐2α), exerts a substantial impact on cellular metabolism under conditions of hypoxia.^5^ EPAS1 has been demonstrated to be upregulated in TC and is associated with the advancement and staging of TC.^6^ Furthermore, EPAS1 was found to stimulate cell proliferation, invasion, and metastasis in various tumors.^7^ Thus, further exploration of the upstream mechanism of EPAS1 in MTC will provide valuable insights into the treatment of MTC. The dysregulation of long noncoding RNA (lncRNA) molecules in tumors has been extensively validated to exhibit either tumor‐suppressive or oncogenic properties, thereby exerting notable influence on tumor initiation and progression.^8^ Among these lncRNAs, zinc finger antisense 1 (ZFAS1) has emerged as a prominent oncogenic factor with widespread implications in the development of diverse tumor types.^9^ Importantly, ZFAS1 expression has been consistently observed to be upregulated in cells of TC.^10^ Furthermore, the elevated expression levels of ZFAS1 in TC have demonstrated a positive association with clinicopathological characteristics and unfavorable prognosis.^11^ Remarkably, mechanistic investigations have convincingly revealed that ZFAS1 actively promotes critical cellular processes including cell proliferation, invasion, and migration in various tumor cells.^12^ Moreover, experimental evidence has established the capacity of ZFAS1 to enhance the expression of EPAS1 in gastric cardia adenocarcinoma.^13^ Collectively, these compelling findings strongly support the hypothesis that ZFAS1 potentially participates in the intricate processes of invasion and metastasis in MTC, possibly through its regulatory influence on EPAS1. It is important to highlight that EPAS1 can undergo degradation via the ubiquitination/proteasomal degradation pathway,^14^ and this posttranslational regulation is frequently influenced by oxygen availability.^15^ Ubiquitin carboxyterminal hydrolase L1 (UCHL1), also known as PGP 9.5, is a deubiquitinating enzyme responsible for removing ubiquitin molecules from target proteins.^16^ UCHL1 has garnered attention as a potential oncogene, and its crucial involvement in regulating tumor cell invasion and metastasis has been extensively documented.^17^ Furthermore, an upregulation of UCHL1 mRNA has been observed in tissues affected by MTC.^18^ The bioinformatics analysis predicted that EPAS1 might be the substrate of UCHL1, but the specific role of UCHL1 in regulating the invasion and metastasis of MTC, as well as its potential interplay with EPAS1 ubiquitination, necessitates further comprehensive investigation.

lncRNAs have been identified to function as competing endogenous RNAs (ceRNAs) capable of regulating target genes by competitively binding to microRNAs (miRNAs). This binding prevents miRNAs from suppressing the expression of their intended mRNA targets, thereby exerting indirect regulatory effects.^19^ In light of this regulatory mechanism, it is plausible that ZFAS1 acts as a ceRNA by sequestering miRNAs that would otherwise target another mRNA. MiR‐214‐3p, known to play a role in tumor cell proliferation, invasion, and migration,^20^ has been found to be downregulated in TC.^21^ The observed downregulation of miR‐214‐3p in TC implies that decreased levels of this miRNA may contribute to the dysregulation of cellular processes associated with increased cell proliferation, enhanced invasion, and heightened migration. Based on these findings, we propose a hypothesis that miR‐214‐3p may participate in the ceRNA network involving ZFAS1, collectively governing the progression of MTC. In addition, our bioinformatics prediction found that miR‐214‐3p and UCHL1 have potential binding sites, but the relationship between them has not been reported.

Based on the aforementioned background, this study is to unravel the upstream molecular mechanism underlying the invasive properties of MTC mediated by EPAS1. We hypothesized that LncRNA ZFAS1 promotes invasion of MTC by upregulating EPAS1 expression via the miR‐214‐3p/UCHL1 axis. The findings of this research are expected to provide novel insights into the pathogenesis of MTC, shedding light on previously unknown molecular targets that may hold therapeutic potential.

## 2 MATERIALS AND METHODS

### 2.1 Clinical samples

A cohort consisting of 8 patients diagnosed with MTC exhibiting persistent or recurrent disease, along with 5 patients with goiter who underwent adenoidectomy serving as controls, was recruited. Prior to specimen collection, the MTC patients had not undergone any preoperative treatments. Human tissue samples obtained were promptly snap frozen in liquid nitrogen and subsequently stored at a temperature of −80°C for subsequent experimental analyses. The study obtained approval from the ethics committee of Qiqihar First Hospital. All participants voluntarily signed a written consent form.

### 2.2 Cell culture

The human MTC cell line of TT and MZ‐CRC‐1 were acquired from the Cell Bank of the Chinese Academy of Sciences and the Cobioer Biosciences Co., Ltd., respectively. The TT cells were cultured in the F12K medium, while the MZ‐CRC‐1 cells were cultured in Dulbecco's Modified Eagle Medium (DMEM) medium. All culture media were supplemented with 10% fetal bovine serum (FBS) and 1% penicillin–streptomycin solution. The cells were maintained in a controlled environment at a temperature of 37°C and a CO_2_ concentration of 5%.

### 2.3 Cell transfection and hypoxic treatment

The miR‐214‐3p mimics, sh‐ZFAS1, sh‐UCHL1, oe‐UCHL1, and their corresponding control sequences (sh‐NCs and oe‐NC), were synthesized and acquired from GenePharma located in Shanghai, China. Lentiviruses utilized in the experiments were also obtained from the same source. The above vectors were transfected into cells using lipofectamine 3000. Following this, the cells were collected for subsequent analysis.

Hypoxia was experimentally induced using InvivO_2_ (Baker Ruskin), a specialized CO_2_/O_2_ incubator/chamber designed for hypoxia research. The cells were subjected to a low oxygen environment by culturing them under conditions of 2% oxygen concentration for a duration of 24 h. The cells in the control group were cultured under normal conditions (95% O_2_, 5% CO_2_).

### 2.4 RNA extraction and quantitative real‐time PCR

Tissue samples and cells were subjected to total RNA extraction using the TRIZOL RNA extraction kit (Tiangen) as per the manufacturer's instructions. The extracted RNA was then reverse‐transcribed into cDNA using the AMV reverse transcription kit. For quantitative real‐time PCR (qRT‐PCR), the SYBR® Green Master Mix Kit was employed following the manufacturer's protocol. The primer sequences used for amplification were listed in Table 1. The relative mRNA expression levels in each experimental group were analyzed using the 2^(−ΔΔCT)^ method. β‐actin was the internal reference of gene expression, and U6 was the internal reference of miR‐214‐3 p expression.

**TABLE 1:** The primer sequences in study (5′–3′).

Gene	Forward	Reverse
lncRNA ZFAS1	ATTGTCCTGCCCGTTAGAGC	ACTTCCAACACCCGCATTCA
EPAS1	ATCATGTGTGTCAACTACGTCC	CTCGAAGTTCTGATTCCCGAAA
miR‐214‐3p	GCGACAGCAGGCACAGACA	AGTGCAGGGTCCGAGGTATT
UCHL1	CAGCATCAGTTGCCTGGGTTA	GCCTAGGGTCGTCTACCAAC
GAPDH	CTGACTTCAACAGCGACACC	GTGGTCCAGGGGTCTTACTC

### 2.5 Western blot assay

Total protein was extracted by RIPA Buffer (#R0278, Sigma‐Aldrich) and determined using the BCA protein assay kit (Solarbio). Subsequently, the protein samples were separated by SDS‐PAGE and transferred onto PVDF membranes. These membranes were then blocked by skim milk and incubated with primary antibodies overnight at 4°C and with the corresponding secondary antibodies after that. Enhanced chemiluminescence reagent was used to visualize the protein bands, and the intensity of each band was analyzed using Image J software. The primary antibodies employed in this study were anti‐EPAS1 (sc‐46691, Santa Cruz) and anti‐UCHL1 (PA5‐29012, Invitrogen). β‐actin was used as the internal control for normalizing the analysis of changes in target protein expression.

### 2.6 Cell viability

Cells were seeded in 96‐well plates and cultured accordingly. The MTT reagent (5 mg/mL, M2003, Sigma Aldrich) was added to the cells and incubated for 6 h at a temperature of 37°C. Following incubation, the supernatant was carefully removed, and the resulting formazan precipitate was dissolved in dimethyl sulfoxide. The absorbance of the dissolved formazan at 490 nm was measured using a microplate reader, providing an indication of cell viability.

### 2.7 EdU staining

EdU staining was conducted using the EdU Imaging Kit (C10310‐1, RiboBio). Briefly, cells were exposed to EdU buffer A for 2 h. Then, cells were fixed by 4% paraformaldehyde for 30 min. A solution containing 2 mg/mL glycine was then added. Subsequently, 0.4% Triton X‐100 was used to permeabilize cells for 10 min. After that, 1× Apollo solution was utilized to stain cells for 30 min under a low‐light condition. Excess staining solution was removed by washing the cells. After that, 1× Hoechst 33342 was applied to visualize the cell nuclei for 30 min, followed by a washing step. The stained cells were observed and imaged using a confocal laser scanning microscope (ZEISS). Finally, the acquired images were analyzed using the ImageJ software (National Institutes of Health).

### 2.8 Transwell invasion and migration assays

Cells were placed into Transwell inserts (353097, Corning) with a seeding density of 8 × 10^4^ cells per well. The bottom of the 24‐well plates was supplemented with 20% FBS. After an incubation period of 24 h, the cells that had migrated to the lower surface of the membrane were stained by 0.1% crystal violet. To ensure representative results, at least three fields were imaged and quantified by Image‐Pro Plus 6.0 software. The invasion assay followed a similar procedure, except that the membrane was precoated with 15% Matrigel (354234, Corning) in DMEM to mimic a barrier.

### 2.9 Immunoprecipitation assay

Cells were subjected to a wash with cold PBS and subsequently lysed by a lysis buffer. The cellular lysates were immunoprecipitated with the anti‐ubiquitin antibody (13–1600, Invitrogen) and the anti‐UCHL1 antibody (PA5‐29012, Invitrogen), with the utilization of the Immunoprecipitation (IP) Kit Dynabeads Protein G (Life Technologies). The IP procedure followed the manufacturer's instructions. Subsequently, western blot analysis was performed.

### 2.10 Immunofluorescence staining

Cells were fixed using 4% paraformaldehyde at room temperature for 15 min and permeabilized by treating with 0.1% Triton X‐100 for 10 min. Next, the coverslips containing the cells were blocked with 3% BSA for 12 h. The coverslips were then incubated with the anti‐EPAS1 antibody (sc‐46691, Santa Cruz) and the anti‐UCHL1 antibody (PA5‐29012, Invitrogen) for 1 h at 4°C. For detection, the samples were probed with the secondary antibody along with DAPI at a dilution of 1:5000 (Roche) and incubated at room temperature for 1 h. Finally, the coverslips were mounted onto slides using Dako Fluorescence Mounting Medium (Agilent), and the fluorescence signals were visualized using fluorescence microscopy (Olympus).

### 2.11 Dual‐fluorescence reporter assay

DIANA (http://diana.imis.athena‐innovation.gr/DianaTools/index.php) and Starbase (https://rnasysu.com/encori/index.php) were utilized to predict the binding sites between lncRNA ZFAS1 and miR‐214‐3p and between miR‐214‐3p and UCHL1, respectively. The wild‐type ZFAS1 cDNA, containing the binding site for miR‐214‐3p, and a mutant ZFAS1 sequence with alterations in the miR‐214‐3p binding site were cloned into the psiCHECK 2.0 vector (Promega). These resulting vectors were named ZFAS1‐WT and ZFAS1‐MUT, respectively. Similarly, the predicted 3′‐untranslated region (UTR) sequence of UCHL1, which interacts with miR‐214‐3p, and mutated sequences within the predicted target sites were synthesized and inserted into the pRL‐TK control vector (Promega). These constructs were designated as UCHL1‐WT and UCHL1‐MUT, respectively. Above plasmids and miR‐214‐3p mimics, along with the mimic NC, were co‐transfected into cells. After 48 h, luciferase assays were performed on cell extracts using the Dual‐Luciferase Assay System (Promega), following the manufacturer's instructions. The data obtained were normalized by calculating the ratio of firefly and Renilla luciferase activities measured.

### 2.12 RNA immunoprecipitation assay

RNA immunoprecipitation (RIP) experiments were conducted using a Magna RIP RNA‐binding protein IP kit (Millipore) following the manufacturer's instructions. Cell lysates were incubated with magnetic beads that were conjugated with IgG or the anti‐Ago2 antibody (Millipore). The immunoprecipitated RNAs were subsequently extracted and then detected by qRT‐PCR.

### 2.13 Nude mice xenograft experiments

The nude mice (5–7 weeks and 18–25 g) were procured from Hunan Slack Jingda Experimental Animal Co., Ltd. and maintained under laboratory conditions with a 12‐h light/dark cycle. TT cells were infected with lentiviruses carrying sh‐NC, sh‐ZFAS1, oe‐NC, or oe‐UCHL1 for a duration of 24 h. Subsequently, a total of 1 × 10^6^ TT cells suspended in 200 μL HBSS were subcutaneously injected into the rear flank of nude mice to induce tumor. To measure the volumes of the tumors, the tumor length (*L*) and tumor width (*W*) were measured every 10 days for 30 days. The volume (*V*) was calculated as the following: *V* (mm^3^) = 0.5 × (*W*)^2^ × (*L*). Then, tumors were dissected and weighed. IHC was performed on tumor tissues using anti‐Ki67 (ab15580, Abcam) and anti‐EPAS1 (sc‐46691, Santa Cruz) antibodies. To investigate lung metastasis, transfected TT cells (1 × 10^6^) from the sh‐NC, sh‐ZFAS1, sh‐ZFAS1+oe‐NC, and sh‐ZFAS1+oe‐UCHL1 groups (*N* = 5) were intravenously injected into nude mice through the tail vein. After a duration of 30 days, lung tissues were collected for subsequent histological analysis using hematoxylin and eosin (H&E, ab245880, Abcam) staining. All animal experiments were conducted following the ethical guidelines and protocols established by the Animal Care and Use Committee of Qiqihar First Hospital.

### 2.14 Statistical analysis

Data analysis was performed using IBM SPSS Statistics 28.0 (IBM Corporation). The Student's *t*‐test was used for the analysis of two groups, whereas one‐way analysis of variance was used for the analysis of three or more groups. A *p*‐value less than 0.05 (*p* < 0.05) was considered statistically significant. The data were presented as the mean ± standard deviation, and all experiments were conducted with a minimum of three biological replicates.

## 3 RESULTS

### 3.1 LncRNA ZFAS1 expression and EPAS1 protein level were upregulated in MTC patients and cells

**FIGURE 1:**
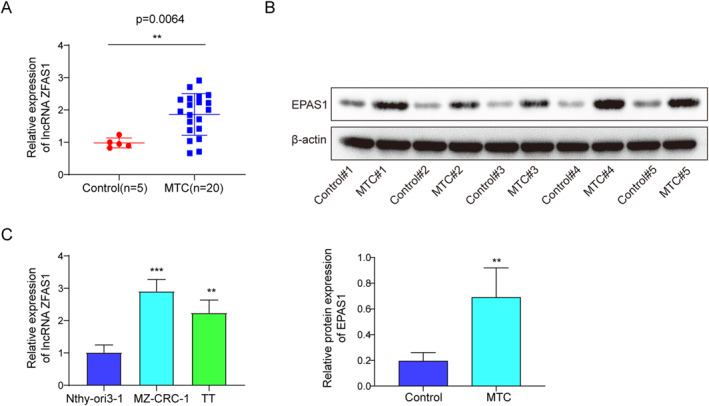
ZFAS1 and EPAS1 protein were upregulated in MTC in vivo and in vitro. A cohort of 8 MTC tissues and 5 healthy thyroid gland tissues was recruited. (A) The expression of ZFAS1 in MTC or normal tissues was assessed using qRT‐PCR. (B) The protein expression level of EPAS1 in MTC or normal tissues was examined by western blot. (C) The expression of ZFAS1 in MTC cells was analyzed using qRT‐PCR. (D) The protein expression level of EPAS1 in MTC cells was evaluated by western blot. **p < 0.01, and ***p < 0.001. MTC, medullary thyroid carcinoma.

LncRNA ZFAS1 was found to facilitate TC,^22^ and EPAS1 expression was upregulated and associated with the pathogenesis of TC.^6^ Therefore, we sought to explore their function in MTC. A total of 8 samples from individuals diagnosed with MTC and 5 samples from individuals with goiter serve as the “Control” group. Experiment results demonstrated that ZFAS1 expression was increased in MTC tissue samples (Figure 1A). Western blot analysis revealed that EPAS1 protein level was significantly elevated in MTC tissue samples compared with normal thyroid tissues (Figure 1B). Subsequently, we found that ZFAS1 level was significantly upregulated in MTC cell lines (MZ‐CRC‐1 and TT) compared to human thyroid follicular cells (Nthy‐ori3‐1) (Figure 1C). These findings demonstrated that ZFAS1 and EPAS1 levels were upregulated in both MTC tissues and cells, suggesting that their aberrant expression may be implicated in the etiology of MTC.

Silencing of ZFAS1 abrogated the proliferation and invasion of MTC cells under hypoxic conditions by decreasing EPAS1 protein level.

**FIGURE 2:**
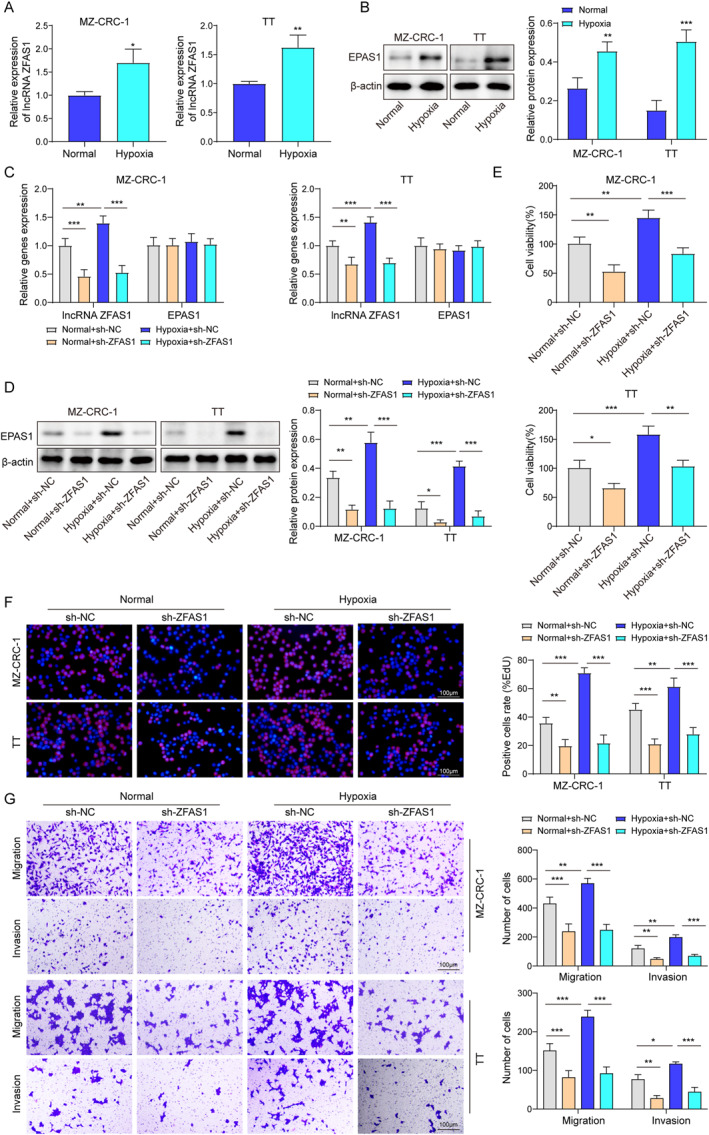
Silencing of ZFAS1 abrogated the proliferation and invasion of MTC cells under hypoxic conditions by decreasing EPAS1 protein level. MTC cells were cultured under hypoxic or normal conditions. (A, B) The expression levels of ZFAS1 and EPAS1 in MTC cells were assessed using qRT‐PCR and western blot assays. Subsequently, cells were transfected with either sh‐NC or sh‐ZFAS1. (C, D) The expression levels of ZFAS1 and EPAS1 in MTC cells were determined using qRT‐PCR and western blot. (E) The viability of MTC cells were evaluated using the MTT assay. (F) The proliferation of MTC cells were measured by the EdU assay. (G) The invasive and metastatic potential of MTC cells were investigated through the Transwell assay. *p < 0.05, **p < 0.01, and ***p < 0.001. MTC, medullary thyroid carcinoma.

It is widely acknowledged that the tumor microenvironment invariably coexists with a hypoxia condition, which drives tumor progression and development.^23^, ^24^ EPAS1 is closely associated with hypoxia conditions, and both EPAS1 and ZFAS1 have been strongly implicated in tumor proliferation and invasion processes.^7^, ^25^ Thus, this study utilized MTC cell lines exposed to hypoxic conditions to simulate the intricate microenvironment characteristic of MTC and further investigate whether ZFAS1 regulated the proliferation and invasion of MTC cells under hypoxic conditions by mediating EPAS1. As shown Figure 2A, hypoxic treatment significantly enhanced ZFAS1 expression. However, hypoxic treatment remarkably increased the protein level of EPAS1 (Figure 2B) while did not affect the expression of EPAS1 mRNA (Figure S1). Furthermore, silencing of ZFAS1 decreased the level of EPAS1 protein but did not influence the expression of EPAS1 mRNA in MTC cells under hypoxic conditions (Figure 2C,D). These findings unveiled that ZFAS1 can modulate EPAS1 in MTC cells at the protein level rather than through the transcriptional mechanism. As shown in Figure 2E–G, under hypoxic conditions, the viability of MTC cells was increased, their proliferation, invasion, and migration capabilities were significantly enhanced. Notably, the targeted silencing of ZFAS1 effectively counteracted these effects induced by hypoxia (Figure 2E–G). The data above suggested that the suppression of ZFAS1 effectively mitigated the hypoxia‐induced proliferation and invasion of MTC cells by downregulating EPAS1 protein levels.

### 3.2 Knocking down of UCHL1 increased EPAS1 ubiquitination by suppressing the interaction between UCHL1 and EPAS1 in the nucleus

**FIGURE 3:**
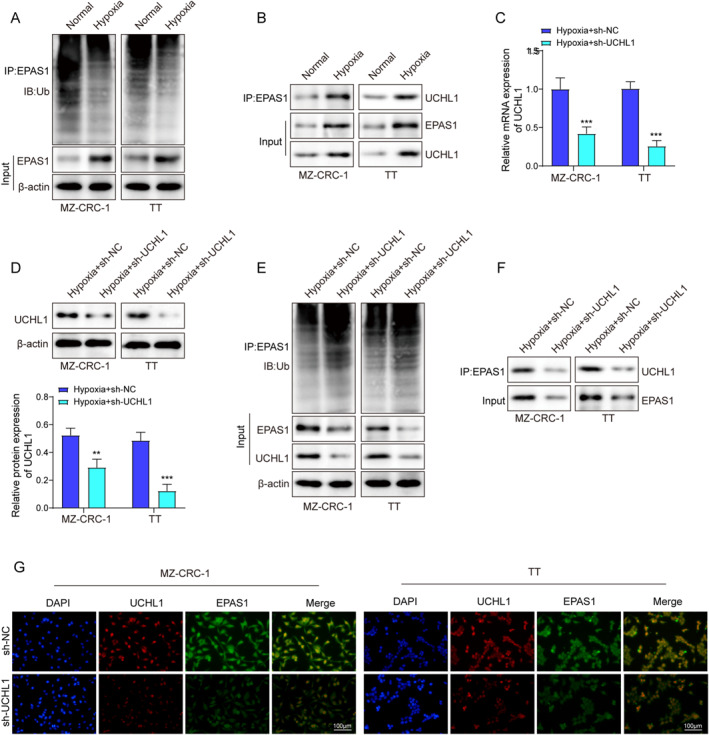
Knocking down of UCHL1 increased EPAS1 ubiquitination by suppressing the interaction between UCHL1 and EPAS1 in the nucleus. (A) IP was utilized to assess the ubiquitination level of EPAS1 in hypoxic or normal MTC cells. (B) IP assay was employed to detect the binding interaction between EPAS1 and UCHL1 in hypoxic or normal MTC cells. (C, D). The expression levels of UCHL1 in hypoxic MTC cells transfected with either sh‐UCHL1 or sh‐NC were determined using qRT‐PCR and western blot. (E) IP was conducted to investigate the ubiquitination level of EPAS1 in hypoxic MTC cells transfected with either sh‐UCHL1 or sh‐NC. (F) IP was utilized to analyze the binding of EPAS1 to UCHL1 in hypoxic MTC cells transfected with either sh‐UCHL1 or sh‐NC. (G) Immunofluorescence was employed to assess the binding of EPAS1 to UCHL1 in hypoxic MTC cells transfected with either sh‐UCHL1 or sh‐NC. *p < 0.05, **p < 0.01, and ***p < 0.001. MTC, medullary thyroid carcinoma.

Since ZFAS1 exerts its regulatory influence on EPAS1 solely at the protein level. Moreover, ubiquitination stands out as an extensively studied process among the plethora of posttranslational modifications, and ZFAS1 was related to ubiquitin‐mediated proteolysis.^11^ Therefore, we hypothesized that ZFAS1 might regulate the ubiquitination of EPAS1 to affect its protein levels. Results showed a notable reduction in the ubiquitination levels of EPAS1 in MTC cells under hypoxic conditions (Figure 3A). Subsequent bioinformatics analyses (UbiBrowser 2.0) predicted the presence of multiple deubiquitinating enzymes that potentially regulate the ubiquitination status of EPAS1. Particularly, previous investigation has reported an upregulation expression of UCHL1 in MTC tissues.^18^ This study also demonstrated that UCHL1 expression was significantly increased in MTC cells under hypoxic conditions, and hypoxia increased the binding interaction between UCHL1 and EPAS1 in MTC cells (Figure 3B). Subsequently, UCHL1 expression was knocked down in MTC cell lines (Figure 3C,D). Knocking down UCHL1 promoted the ubiquitin‐mediated degradation of EPAS1 in hypoxia‐induced MTC cells (Figure 3E). Furthermore, UCHL1 knockdown inhibited the hypoxia‐induced binding interaction between UCHL1 and EPAS1 (Figure 3F) and reduced the colocalization of UCHL1 and EPAS1 within the nucleus induced by hypoxia (Figure 3G). These findings have elucidated that the reduction of UCHL1 expression increased the ubiquitination of EPAS1.

### 3.3 LncRNA ZFAS1 sponged miR‐214‐3p to regulate UCHL1

**FIGURE 4:**
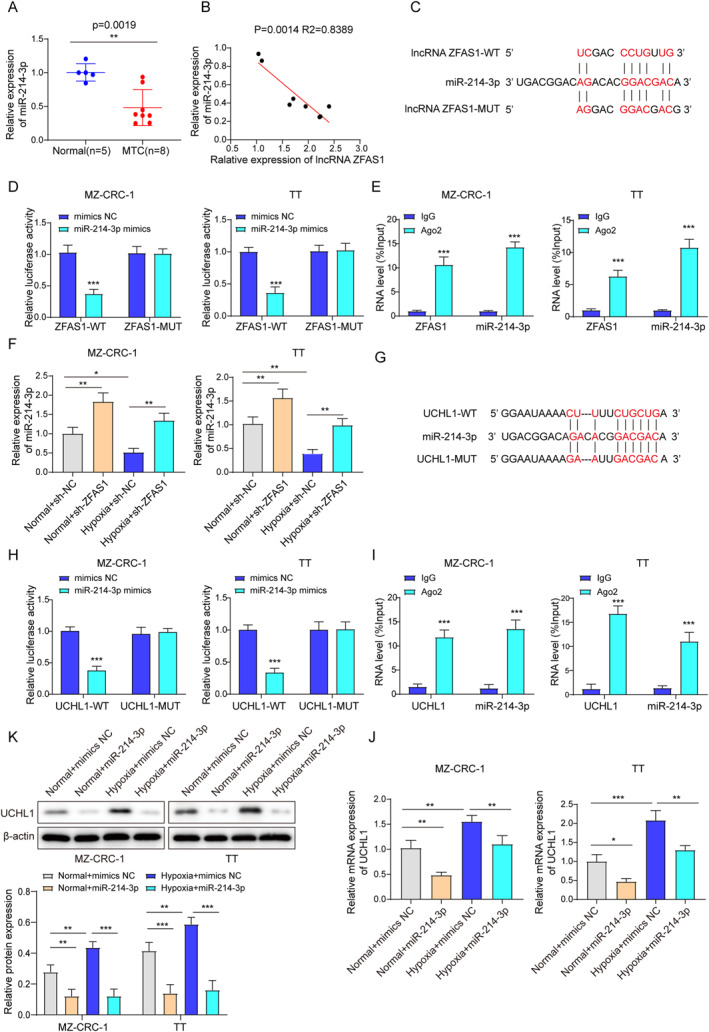
LncRNA ZFAS1 sponged miR‐214‐3p to regulate UCHL1 (A). qRT‐PCR was utilized to measure miR‐214‐3p expression in MTC and normal tissues. (B) Correlation analysis was performed to investigate the association between miR‐214‐3p expression levels and those of ZFAS1/UCHL1 in MTC patient tissues. (C, G) Starbase was used to predict the potential binding of ZFAS1/UCHL1 to miR‐214‐3p. (D, H) A dual‐luciferase reporter assay was conducted to validate the binding of ZFAS1/UCHL1 mRNA to miR‐214‐3p. (E, I) An RNA immunoprecipitation assay was implemented to further verify the binding of ZFAS1/UCHL1 mRNA to miR‐214‐3p. (F) qRT‐PCR was utilized to quantify the expression of miR‐214‐3p in hypoxic/normal MTC cells transfected either with sh‐NC or sh‐ZFAS1. (F, J) qRT‐PCR was used to examine the expression of miR‐214‐3p and UCHL1 mRNA in hypoxic/normal MTC cells transfected with NC or miR‐214‐3p mimics. (K) Western blot was used to measure the UCHL1 protein level in hypoxic/normal MTC cells transfected with NC or miR‐214‐3p mimics. *p < 0.05, **p < 0.01, and ***p < 0.001. MTC, medullary thyroid carcinoma.

LncRNA has the ability to modulate the expression of their target mRNAs as ceRNAs, and miR‐214‐3p expression was reported to be downregulated in TC,^21^ which was further verified in our study (Figure 4A). Additionally, the results showed that miR‐214‐3p was positively related to both ZFAS1 and UCHL1 (Figure 4B). Furthermore, miR‐214‐3p was predicted to bind with both ZFAS1 and UCHL1 (Figure 4C,G). The dual‐luciferase reporter gene assay revealed that miR‐214‐3p mimics significantly suppressed luciferase activity in the ZFAS1‐WT or UCHL1‐MUT but not in the ZFAS1‐MUT or UCHL1‐MUT (Figure 4D,H). Additionally, the RIP assays demonstrated the enrichment of ZFAS1 and miR‐214‐3p in the Ago2 complex (Figure 4E), indicating their interaction and association with the RNA‐induced silencing complex. UCHL1 and miR‐214‐3p were also found to be enriched in the Ago2 complex (Figure 4I). A further study found that hypoxia suppressed the expression of miR‐214‐3p while ZFAS1 silencing elevated its expression (Figure 4F), and hypoxia increased the expression of UCHL1 mRNA and protein, which was reversed by miR‐214‐3p overexpression (Figure 4J,K). These findings demonstrated that ZFAS1 may regulate UCHL1 by competitively interacting with miR‐214‐3p, thereby modulating its activity and expression.

### 3.4 Overexpression of UCHL1 reversed the suppression of MTC cell proliferation and invasion under hypoxic conditions caused by ZFAS1 silencing

**FIGURE 5:**
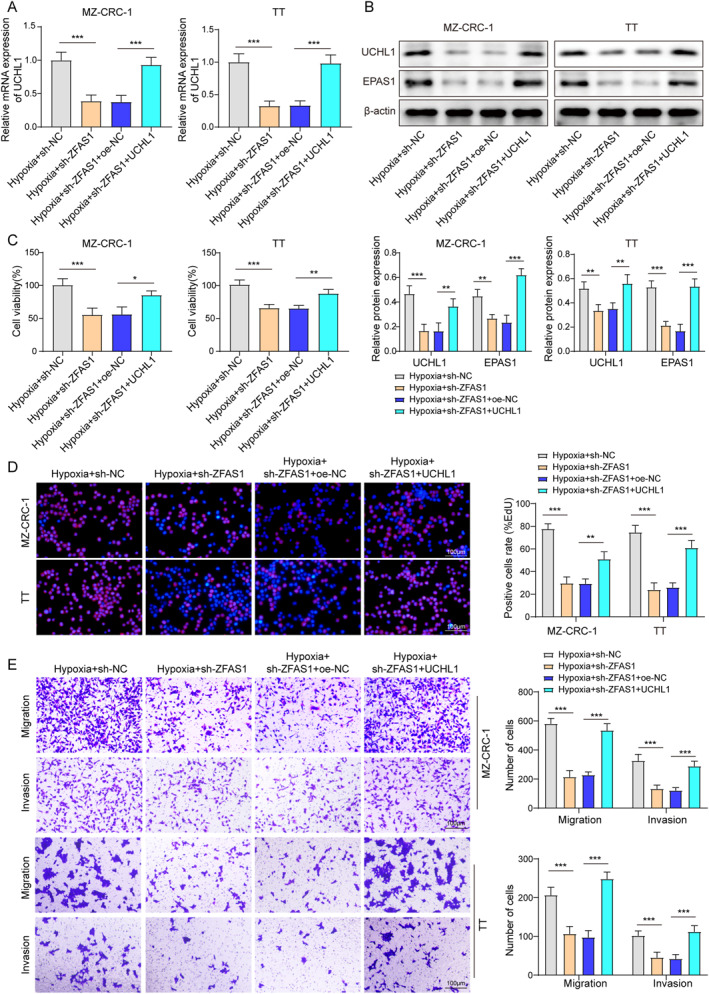
Overexpression of UCHL1 reversed the suppression of MTC cell proliferation and invasion under hypoxic conditions caused by ZFAS1 silencing. MTC cells were transfected with sh‐NC/sh‐ZFAS1/oe‐NC/oe‐UCHL1 and cultured under hypoxic or normal conditions. (A, B) qRT‐PCR and western Blot were used to assess the expression of UCHL1 and EPAS1. (C) The cell viability of MTC cells was quantified via the MTT assay. (D) EdU staining was utilized to measure the cell proliferation. (E) Transwell assay was employed to detect the cell invasion and migration abilities. *p < 0.05, **p < 0.01, and ***p < 0.001. MTC, medullary thyroid carcinoma.

To elucidate the mechanistic role of UCHL1 in the regulation of cellular proliferation and invasion mediated by ZFAS1 in MTC cells, this investigation established MTC cell lines with ZFAS1 knockdown and UCHL1 overexpression in a hypoxic microenvironment. The findings exhibited that depletion of ZFAS1 inhibited the expression of UCHL1 mRNA and protein, whereas increase of UCHL1 expression counteracted the inhibitory effect of ZFAS1 silencing on UCHL1 mRNA and protein expression (Figure 5A,B). Further studies demonstrated that the overexpression of UCHL1 reversed the suppressive effects on cellular viability, proliferation, and invasion of MTC cells caused by ZFAS1 silencing (Figure 5C–E). These results imply that ZFAS1 silencing inhibits cellular proliferation and invasion by suppressing the expression of UCHL1.

### 3.5 Silencing of ZFAS1 repressed tumor formation and metastasis of MTC in vivo via modulation of the miR‐214‐3p/UCHL1/EPAS1 axis

**FIGURE 6:**
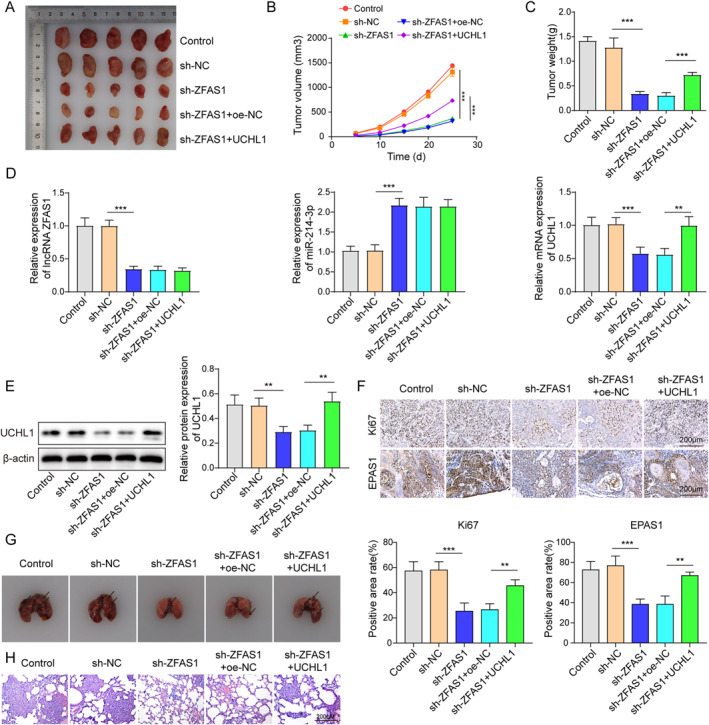
Silencing of ZFAS1 repressed tumor formation and metastasis of MTC in vivo via modulation of the miR‐214‐3p/UCHL1/EPAS1 axis. (A–C) Assessment of tumor volume, growth curve, and tumor weight in xenograft mouse models. (D, E) qRT‐PCR and western blot analysis were employed to evaluate the expression levels of ZFAS1, miR‐214‐3p, and UCHL1. (F) IHC analysis was utilized to assess the expression of Ki67 and EPAS1. (G–H) Acquisition of lung metastasis photographs and histopathological examination by H&E staining to identify pathological alterations. *p < 0.05, **p < 0.01, and ***p < 0.001. qRT‐PCR and western blot analysis were used to assess the expression levels of EPAS1 under hypoxic or normal conditions. EPAS1, endothelial PAS domain‐containing protein 1.

Finally, this study proceeded to validate the role of ZFAS1/miR‐214‐3p/UCHL1/EPAS1 in MTC in vivo by establishing a subcutaneous xenograft model of MTC in nude mice. Experimental results demonstrated that the silencing of ZFAS1 significantly inhibited tumor growth, while the overexpression of UCHL1 abolished this inhibitory effect (Figure 6A–C). Moreover, the knockdown of ZFAS1 promoted the expression of miR‐214‐3p, while concurrently suppressing the expression of UCHL1. Conversely, the overexpression of UCHL1 reversed these effects mediated by ZFAS1 (Figure 6D,E). IHC assays revealed that the silencing of ZFAS1 reduced Ki67 and EPAS1 expression, whereas the increase of UCHL1 overturned these inhibitory effects induced by ZFAS1 knockdown (Figure 6F). Finally, ZFAS1 knockdown was observed to suppress MTC lung metastasis, while the upregulation of UCHL1 mitigated this suppressive effect (Figure 6G,H). Results demonstrated that the silencing of ZFAS1 effectively inhibited MTC tumor progression and lung metastasis by upregulating miR‐214‐3p and downregulating UCHL1, suggesting that ZFAS1 can attenuate MTC progression through the miR‐214‐3p/UCHL1/EPAS1 pathway.

## 4 DISCUSSION

The incidence of TC has shown a remarkable rise globally in the past 4 decades.^26^ Among the different subtypes, MTC accounts for approximately 13.4% of TC‐related deaths.^27^ MTC is characterized by its high invasiveness, leading to frequent metastasis and relapse^28^ and challenges in the management and treatment of MTC. Considering the limited treatment options available for MTC, investigating its pathogenesis assumes paramount importance.

Emerging evidence has revealed that a substantial proportion of lncRNAs play either promoting or inhibitory roles in the pathogenesis of TC, highlighting their potential as diagnostic and prognostic biomarkers for TC.^29^ Notably, several lncRNAs have been reported to facilitate the proliferation, invasion, and migration of TC cells, thereby promoting disease progression.^28^ In our study, we demonstrated the upregulation of lncRNA ZFAS1 in MTC. Although a previous investigation by Tong et al. reported increased ZFAS1 expression in papillary thyroid carcinoma (PTC), the molecular mechanism of ZFAS1 in MTC has not been explored.^10^ Here, we demonstrated that silencing ZFAS1 leads to a significant reduction in the proliferation and invasion of MTC cells under hypoxic conditions. These observations strongly indicate that ZFAS1 could serve as a promising therapeutic target for the treatment of MTC.

Hypoxia is recognized as a crucial factor contributing to the invasive and metastatic behavior of TC cells.^4^ EPAS1 serves as an alpha subunit of the HIF, playing a crucial role in cellular responses to hypoxia by binding to hypoxia‐response elements in the promoter regions of target genes.^30^ Previous research conducted by Zhang et al. demonstrated elevated mRNA expression of EPAS1 in PTC, leading to the inhibition of PTC development.^6^ However, the study did not delve into the underlying mechanisms responsible for the aberrant expression of EPAS1 or explore whether EPAS1 exhibits similar upregulation in other subtypes of TC. In our study, we observed upregulation of EPAS1 in MTC. Notably, silencing of ZFAS1 resulted in an increase in the protein level of EPAS1. Intriguingly, the mRNA expression of EPAS1 remained unaffected during this process, suggesting that ZFAS1 might regulate EPAS1 at the posttranslational level. These findings contribute to our understanding of the intricate regulatory networks involved in MTC and shed light on the role of ZFAS1 in modulating the expression and function of EPAS1.

Substrate protein ubiquitination represents an intricate and tightly regulated process, wherein ubiquitin molecules are covalently attached to specific lysine residues on the target protein.^31^ Importantly, protein ubiquitination is a reversible phenomenon, and the involvement of deubiquitinases (DUBs) ensures the removal of ubiquitin moieties from substrate proteins, thereby rescuing them from proteasomal degradation and preventing their untimely breakdown.^32^ Among the various DUBs, UCHL1 has garnered considerable attention due to its implication in diverse cancer types, including MTC.^18^ Notably, Takano et al. reported an observation of increased UCHL1 mRNA expression specifically in MTC tissues.^18^ However, a comprehensive exploration of the specific functions of the upregulated UCHL1 in MTC, as well as the underlying mechanisms regulating its aberrant gene regulation, was not undertaken in their study. In this present study, we elucidated the upregulation of UCHL1 in MTC cells under hypoxic conditions, thereby providing novel insights into the functional significance of UCHL1 upregulation in MTC. Furthermore, our investigation successfully confirmed that the silencing of UCHL1 led to an increase in the ubiquitination of EPAS1. These compelling findings not only illuminate the intricate mechanisms underlying UCHL1‐mediated deubiquitination of EPAS1 but also contribute to a deeper understanding of the dysregulation of EPAS1 in the context of MTC. In addition to UCHL1, other DUBs may also have the potential to modulate the ubiquitination process of EPAS1, as depicted in Figure 3B. These DUBs include USP37, USP7, USP9X, USP28, USP33, USP2, USP4, USP5, USP45, and BAP1. Notably, USP37 has been identified as a facilitator of EPAS1 deubiquitination in kidney cancer.^33^ However, the involvement of USP37 and other DUBs in the regulation of EPAS1 specifically within the context of MTC needs further investigation. Moreover, it is crucial to recognize that apart from ubiquitination, a plethora of posttranslational modifications, such as phosphorylation, SUMOylation, and acetylation, play significant roles in the intricate regulation of proteins. Therefore, there might be additional posttranslational modification mechanisms contributing to the regulation of EPAS1 protein in the context of MTC.

The ceRNA network involving lncRNA, miRNA, and mRNA has been shown to function as both tumor suppressors and oncogenic factors in the context of TC.^34^ Within this ceRNA network, the 5′ end of miRNAs contains nucleotides that have the ability to bind to multiple mRNAs and lncRNAs through specific sites called miRNA response elements, typically located in the 3′ UTRs of the target RNAs. Consequently, miRNAs can exert posttranscriptional regulation by modulating the expression of these targeted RNAs.^35^ In this study, we discovered the involvement of the ZFAS1/miR‐214‐3p/UCHL1 network in the EPAS1‐mediated invasion of MTC. Mechanistically, ZFAS1 functions as a ceRNA by competitively binding with miR‐214‐3p and preventing its interaction with UCHL1 mRNA. It is worth noting that previous studies have reported the direct binding of ZFAS1 to EPAS1, leading to the upregulation of EPAS1 expression in gastric cancer.^13^ Hence, it is possible that the regulation of EPAS1‐mediated invasion in MTC could occur either through direct regulation of ZFAS1 or by indirect regulation of the ZFAS1/miR‐214‐3p/UCHL1 network.

In conclusion, this study demonstrated that the lncRNA ZFAS1 promotes EPAS1‐mediated invasion in MTC through the ceRNA network of ZFAS1/miR‐214‐3p/UCHL1 (see Graphical abstract). These findings underscore the significance of ZFAS1 as a potential therapeutic target for managing MTC. Targeting ZFAS1 holds promising implications for the development of innovative therapeutic strategies aimed at inhibiting MTC invasion and improving patient outcomes.

## AUTHOR CONTRIBUTIONS

**Wenjing Chen**: Conceptualization; writing—original draft. **Shaoqing Wang**: Formal analysis. **Dongmei Wei**: Investigation. **Lili Zhai**: Resources. **Li Liu**: Data curation. **Chunlei Pan**: Methodology. **Zhongshu Han**: Supervision. **Huiming Liu**: Writing—review and editing. **Wei Zhong**: Visualization; funding acquisition. **Xin Jiang**: Validation; project administration.

## CONFLICT OF INTEREST STATEMENT

The authors declare that they have no conflict of interest.

## ETHICS STATEMENT

The study obtained approval from the ethics committee of Qiqihar First Hospital. All participants voluntarily signed a written consent form. All animal experiments were conducted following the ethical guidelines and protocols established by the Animal Care and Use Committee of Qiqihar First Hospital.

## CONSENT FOR PUBLICATION

All participants voluntarily signed a written consent form.

## Supporting information

Figure S1

## Data Availability

The datasets generated during and/or analyzed during the current study are available from the corresponding author on reasonable request.
