# Supplementary material for: LncRNA ZFAS1 promotes invasion of medullary thyroid carcinoma by enhancing EPAS1 expression via miR‐214‐3p/UCHL1 axis
Source: J Cell Commun Signal. 2024 Apr 12;18(2):e12021. doi: 10.1002/ccs3.12021 (PMC11208124; doi:10.1002/ccs3.12021)
Supplement: Supplementary file 1 — Figure S1 [file CCS3-18-e12021-s001.docx]

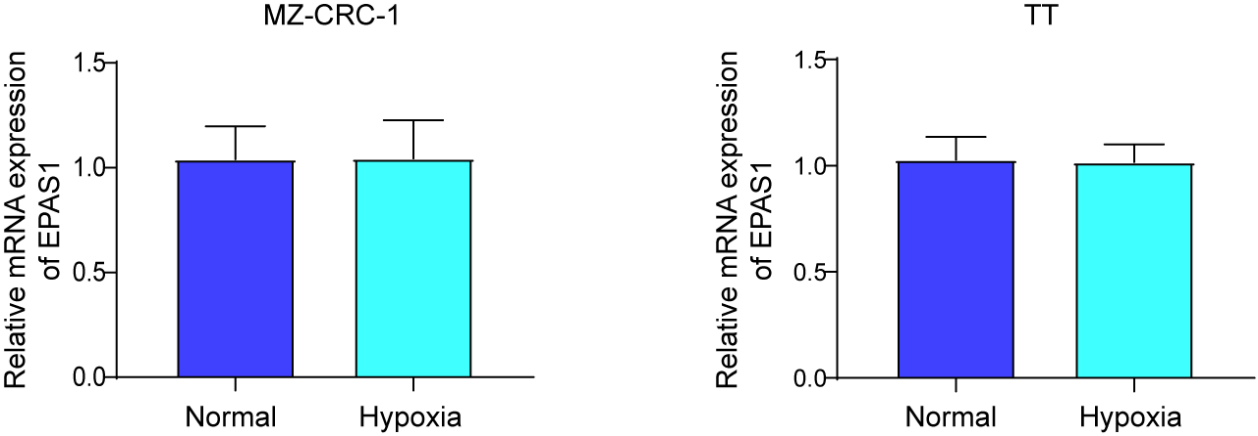


**Fig S1. Hypoxic treatment did not affect the expression of EPAS1 mRNA.**

qRT-PCR detected the expression of EPAS1 mRNA under hypoxic or normal conditions in MTC cells.
